# Immunogenicity and safety of high-dose versus double-dose quadrivalent inactivated influenza vaccine in adult kidney transplant recipients: a randomized controlled trial (2023)

**DOI:** 10.1016/j.lansea.2026.100873

**Published:** 2026-09-25

**Authors:** Thanachote Arayanumas, Kobporn Boonnak, Surasak Kantachuvesiri, Sarinya Boongird, Sasisopin Kiertiburanakul, Jackrapong Bruminhent

**Affiliations:** a Department of Medicine, Faculty of Medicine Ramathibodi Hospital, Mahidol University, Bangkok, Thailand; b Department of Immunology, Faculty of Medicine Siriraj Hospital, Mahidol University, Bangkok, Thailand; c Division of Nephrology, Department of Medicine, Faculty of Medicine Ramathibodi Hospital, Mahidol University, Bangkok, Thailand; d Ramathibodi Excellence Center for Organ Transplantation, Faculty of Medicine Ramathibodi Hospital, Mahidol University, Bangkok, Thailand; e Division of Infectious Diseases, Department of Medicine, Faculty of Medicine Ramathibodi Hospital, Mahidol University, Bangkok, Thailand

**Keywords:** Influenza, Immunocompromised, Vaccination, Transplantation

## Abstract

**Background:**

Solid organ transplant (SOT) recipients are at increased risk of influenza-related complications and often exhibit suboptimal vaccine responses. While both high-dose (HD) and double-dose (DD) quadrivalent inactivated influenza vaccines (QIIV) aim to enhance immunogenicity compared to the standard dose, access to HD QIIV may be limited in resource-constrained settings. We evaluated whether DD QIIV could serve as a practical alternative.

**Methods:**

We conducted open-label randomized controlled trial comparing the 2023 Southern Hemisphere HD QIIV containing 60 μg of antigen per strain with a double dose of standard QIIV containing 30 μg per strain in adult kidney transplant (KT) recipients. Pre- and 4-week post-vaccination sera were tested using strain-specific hemagglutination inhibition assays. Safety was also assessed.

**Findings:**

Eighty-eight KT recipients were randomized and 84 were eligible for analysis (42 per group); 59.5% were male, with a mean age of 46 ± 11 years, and 60.7% received deceased donor kidneys. Seroconversion (SC) to ≥1 strain was significantly higher in the HD group (95.2%) than in the DD group (69%) (P = 0.002). The absolute difference in response rate was 0.26 (95% CI, 0.12–0.40). SC rates were numerically higher in the HD group for all strains. Injection site pain and myalgia were less common in the HD group, with P = 0.040 and P = 0.043, respectively.

**Interpretation:**

HD QIIV demonstrated more favorable immunogenicity and tolerability than DD QIIV. These findings suggest that HD QIIV may be a preferable option for this population when available.

**Funding:**

This study was supported by the Research grant from Faculty of Medicine Ramathibodi Hospital, 10.13039/501100004156Mahidol University, Bangkok, Thailand.

**Research in context**

**Evidence before this study**

Influenza is a common cause of respiratory tract infection, with greater severity in immunocompromised populations, including solid organ transplant (SOT) recipients receiving immunosuppressive therapy, who are at increased risk of lower respiratory tract disease and mortality. Although vaccination is the cornerstone of prevention, immune responses are often suboptimal in these patients. We searched PubMed for studies published up to May 2023 using the terms “influenza vaccine,” “high-dose,” “double-dose,” “standard-dose,” and “solid organ transplant.” High-dose (HD) and double-dose (DD) strategies have been developed to enhance immunogenicity compared with the standard dose (SD); however, direct comparisons between a double dose of standard quadrivalent inactivated influenza vaccine (QIIV) administered at a single visit and high-dose formulations remain scarce, particularly in settings with limited access to HD vaccines.

**Added value of this study**

This study assessed the immunogenicity and safety of HD (60 μg) versus DD (15 μg × 2 at a single visit) QIIV in kidney transplant recipients. Seroconversion to at least one influenza strain was higher in the HD group (95.2%) than in the DD group (69%), with consistently higher responses across individual strains. Adverse events were mild and self-limited, with fewer local and systemic reactions observed in the HD group. These findings provide direct comparative evidence suggesting higher immunogenicity with acceptable safety for HD QIIV in this population.

**Implications of all the available evidence**

These findings indicate that HD QIIV is associated with improved immunogenicity and an acceptable safety profile in SOT recipients receiving immunosuppressive therapy. Where available, it may be considered as an option for this immunocompromised population.

## Introduction

Influenza infection is known to be an important cause of morbidity and mortality around the world especially in solid organ transplant (SOT) recipients. Complications of influenza infection, including bacterial pneumonia, appear to be common in this patient population. In a multi-center study of SOT recipients, hospitalization rates for influenza were reported at about 57–70%, with ICU admission needed in 13–20%, and overall mortality 4–8%.^1^ Therefore, prevention of influenza infection is strongly recommended. Although annual influenza vaccination is recommended for all SOT recipients, the immune response to vaccination is often suboptimal because of lifelong immunosuppression.^1^^,^^2^ Several strategies have been proposed to enhance vaccine immunogenicity in immunocompromised patients, including booster dosing, alternative delivery routes (e.g., intradermal rather than intramuscular), use of adjuvanted or recombinant vaccine platforms, and increasing the antigen dose.

The American Society for Transplantation/IDCOP Guideline (2019) recommends that high-dose (HD) or one-month boosted standard-dose (SD) influenza vaccination provides greater immunogenicity than a single SD, and may be preferred over standard dosing.^3^ However, a two-visit requirement for vaccination may reduce completion rates. While the HD influenza vaccine, containing 60 μg of each circulating strain—representing a four-fold increase compared to the SD—has been approved for use since 2022. Previous studies have shown that both HD and double-dose (DD) inactivated influenza vaccines elicit a stronger immune response in SOT recipients compared to SD vaccines.^4^, ^5^, ^6^ However, a direct comparison between HD and DD quadrivalent inactivated influenza vaccines (QIIV) in immunocompromised populations—such as SOT recipients, people living with HIV, and patients with cancer receiving chemotherapy—has not yet been explored. As access to HD QIIV may be limited in resource-constrained settings due to financial incompatibility or unavailable, whereas DD QIIV—achieved by administering two SD units—offers an immediate alternative. Therefore, the objective of this study was to provide a direct comparison of the immunogenicity and safety of HD versus DD QIIV in adult kidney transplant (KT) recipients. By evaluating whether DD QIIV serves as a practical alternative to HD QIIV, this trial aims to inform clinical guidelines and improve vaccine equity for vulnerable transplant populations worldwide.

## Methods

This single center, open-label, randomized controlled trial was conducted from June to December 2023 at Ramathibodi Hospital, Mahidol University, Bangkok, Thailand. We enrolled adult KT recipients (age ≥15 years old) who have been maintained on at least 3 months after transplant and stable in terms of allograft function and immunosuppressive regimen. Patients with concurrent infection or rejection, a history of severe allergic reaction to influenza vaccine, or receiving influenza vaccination within 6 months were excluded (Supplementary Protocol).

Baseline characteristics including age, sex, time from transplant, time of vaccination post-transplant, previous year influenza vaccination, type of immunosuppressive regimens, absolute lymphocyte count [cells/μl] were collected. Participants were randomized to receive either HD or DD influenza vaccine in a 1:1 ratio. Randomization was performed using a computer-generated random block method. Both vaccines contained the same 4 influenza antigens of the 2023 southern hemisphere; A/Sydney/5/2021 (H1N1)pdm09-like virus, A/Darwin/9/2021-like virus (H3N2), B/Austria/1359417/2021(B/Victoria lineage)-like virus and B/Phuket/3073/2013 (B/Yamagata lineage)- like virus. The HD vaccine contained 60 μg of antigen per strain (Efluelda, Sanofi), while the DD vaccine consisted of two standard doses administered in separate arms during the same visit, totaling 30 μg per strain (Vaxigrip Tetra, Sanofi).

Immunogenicity was assessed by the measurement of hemagglutinin-inhibition (HAI) titers using the hemagglutinin-inhibition assay performed in the same batch at the Department of Immunology, Faculty of Medicine Siriraj Hospital, Mahidol University, Bangkok, Thailand, following the standardized WHO methods to assess strain-specific immune responses before vaccination and 4 weeks post-vaccination. Briefly, serum was incubated with standardized amounts of influenza antigens followed by addition of a suspension of 0.5% red blood cells. Geometric mean titers (GMTs) were determined by doubling dilutions of antibody starting from an initial dilution of 1:8 to a final dilution of 1:1024. Samples with a negative result were assigned a titer of 1:4. Internal positive (same highly positive serum) and negative controls were used.

All participants were monitored for solicited AEs within 30 min after vaccination and on day-3 and 7 by telephone. In addition, unsolicited AEs were documented by participants in the provided diaries.

The primary endpoint of this study was the evaluation of immunogenicity, determined by the proportion of participants who achieved seroconversion to at least one of the four influenza strains included in the vaccine. Seroconversion was assessed using the HAI assay, comparing antibody titers before vaccination and at 4 weeks post-vaccination. Seroconversion was defined as an HAI titer of ≥1:40 in individuals whose baseline titer was <1:10, or a ≥4-fold increase in those with a baseline titer ≥1:10. Secondary immunogenicity outcomes included the geometric mean titers (GMTs), seroprotection rates, seroconversion rates, and seroconversion factors for each vaccine component. Seroprotection was defined as an HAI titer of at least 1:40, following established criteria for evaluating influenza vaccine response. GMTs were calculated as a summary measure of antibody levels across the cohort. Additional secondary outcomes included the incidence of solicited and unsolicited adverse events, both local and systemic. Adverse events were also graded using standardized toxicity criteria: Grade 1 (mild) were transient and did not interfere with daily activities; Grade 2 (moderate) caused some limitation and may require minimal medical management; and Grade 3 (severe) resulted in marked limitation, required medical intervention, or necessitated hospitalization (Supplementary Figs. S1 and S2).^7^

### Statistical analyses

The sample size was determined based on previous studies that used seasonal influenza vaccine in SOT recipients which had indicated a response rate of HD vaccine approximately 80% to at least 1 strain and 50% at DD vaccine, respectively.^4^^,^^5^ The sample size was calculated using an alpha of 0.05 and a power of 80%, yielding a requirement of 40 participants per group. After accounting for an anticipated 10% dropout rate, 44 evaluable patients were required in each study group. The immunogenicity analysis was only performed in those who received a vaccine dose and returned for follow-up serum (per-protocol population).

Baseline demographics and clinical characteristics were analyzed using descriptive statistics. The continuous data were test normality by Shapiro–Wilk normality test and reported as mean and standard deviation (SD) or median and inter quartile range (IQR) with non-normality data. The X^2^ test and Fisher exact test were performed to compare categorical variables as appropriate. The Mann–Whitney U test and Wilcoxon matched-pairs signed-rank test were performed to compare continuous variables between and within the groups, respectively. For multivariate analysis, logistic regression analysis was used to identify predictors of seroconversion to at least one strain. Statistical analyses were performed using STATA version 18 (Stata Crop., College Station, Texas, USA). P values of <0.05 were considered statistically significant.

### Ethics statement

The Institutional Review Board of the Faculty of Medicine, Ramathibodi Hospital, Mahidol University, Bangkok, Thailand, approved the study protocol (approval number: MURA2023/309). The study was conducted following the principles laid out in the Declaration of Helsinki and was registered with the Thai Clinical Trials Registry (TCTR20230510005, 10/05/2023). Informed consent was obtained from all subjects.

### Role of the funding source

The funders had no role in study design, data collection, data analysis, data interpretation, or writing of this manuscript.

## Results

We screened 97 participants during June to December 2023. Among those, 9 patients were excluded due to several reasons including declined to participate (n = 4) and already vaccinated (n = 5). Therefore, 88 eligible KT recipients were randomized to receive either the HD or DD vaccine, and 84 participants (42 in each group) were included in the final analysis (Fig. 1). The baseline characteristics of the participants from each group were well balanced (Table 1). Among them, 50 (59.5%) were male, with a mean age (SD) of 46 (11) years old, and 50 (60.7%) participants received kidneys from deceased donors. Immunosuppressive regimens included mycophenolate mofetil (65.5%), tacrolimus (77.1%), cyclosporine (22.6%), and prednisolone (100%).

**Fig. 1:**
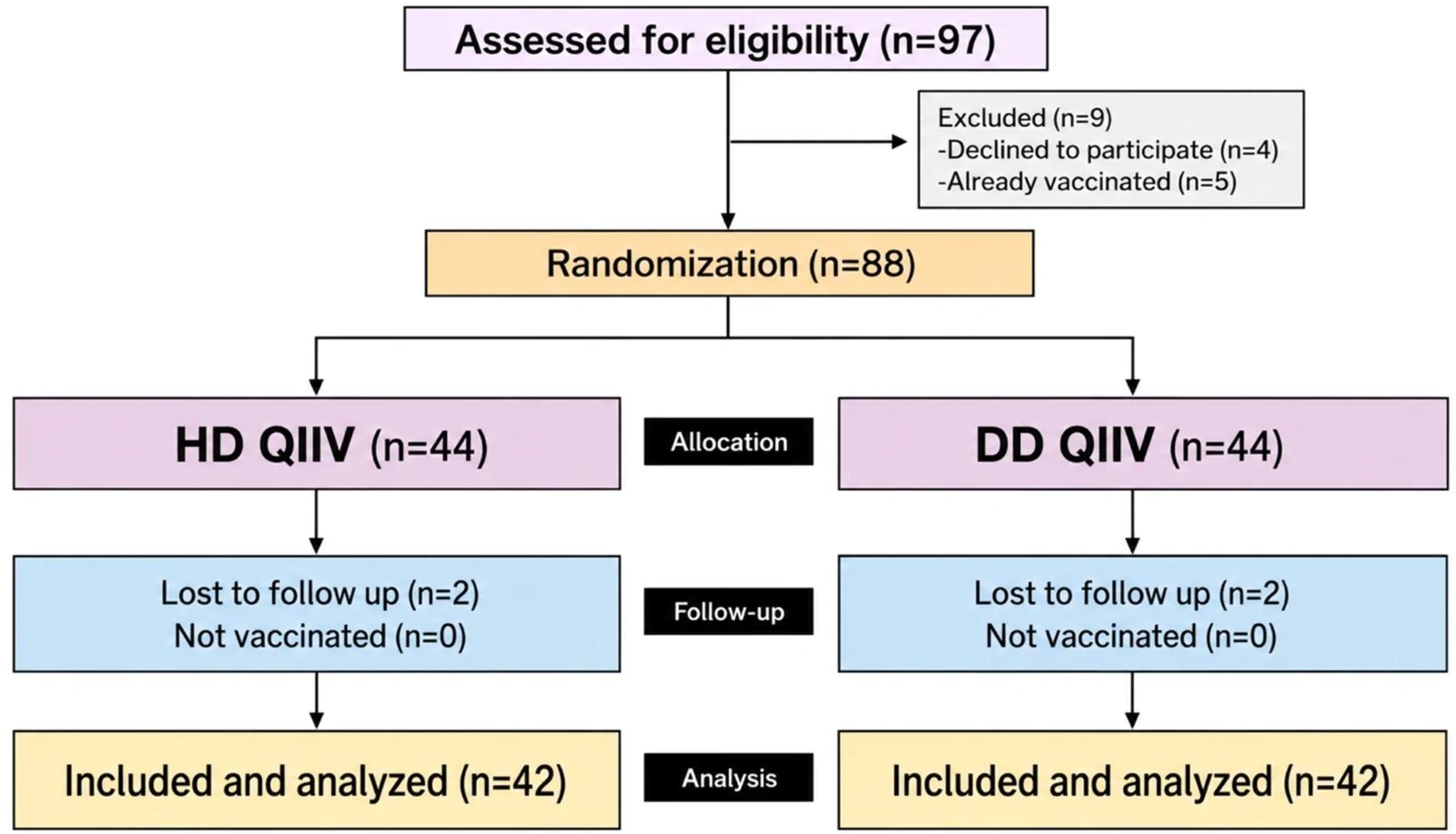
Flowchart of the study.

**Table 1:** Clinical characteristics of the study participants.

Clinical characteristics	Total (n = 84)	HD QIIV (n = 42)	DD QIIV (n = 42)
Age [years], mean (SD)	45.8 (11.1)	45.3 (10.0)	46.2 (12.1)
Male sex, n (%)	50 (59.5)	26 (61.9)	24 (57.1)
Time from transplant [months], median (IQR)	62.5 (31.5, 105.5)	65.5 (36.0, 95.0)	55.5 (31.0, 115.0)
Vaccinate after 1 year post-transplant, n (%)	78 (92.9)	39 (92.9)	39 (92.9)
Previous rejection	4 (4.8)	2 (4.8)	2 (4.8)
Type of transplant, n (%)			
DDKT	51 (60.7)	27 (64.3)	24 (57.1)
LRKT	33 (39.3)	15 (35.7)	18 (42.9)
Immunosuppressants, n (%)			
Tacrolimus, n (%)	64 (77.1)	35 (85.4)	29 (69.0)
Cyclosporine, n (%)	19 (22.6)	8 (19.0)	11 (26.2)
Mycophenolate mofetil, n (%)	55 (65.5)	24 (57.1)	31 (73.8)
Mycophenolic sodium, n (%)	18 (21.4)	12 (28.6)	6 (14.3)
Everolimus, n (%)	6 (7.1)	4 (9.5)	2 (4.8)
Sirolimus, n (%)	2 (2.4)	1 (2.4)	1 (2.4)
Prednisolone, n (%)	84 (100.0)	42 (100)	42 (100)
Absolute lymphocyte count [cells/μl], median (IQR)	1945.0 (1500.0, 2400.0)	2000.0 (1500.0, 2400.0)	1800.0 (1500.0, 2200.0)
Previous year influenza vaccination^a^, n (%)	70 (83.3)	33 (78.6)	37 (88.1)

Data apply to all patients who received study vaccine.

HD QIIV, high-dose quadrivalent inactivated influenza vaccine; DD QIIV, double-dose quadrivalent inactivated influenza vaccine; SD, standard deviation; IQR, interquartile range; DDKT, deceased donor kidney transplant; LRKT, living related kidney transplant; n, number.

a Based on self-report at the time of enrollment.

Among the 88 enrolled patients in the HD and DD vaccine groups, all patients received vaccine; However, four patients (two in each group) were excluded from the immunogenicity analysis due to loss to follow-up (Fig. 1). Therefore, the immunogenicity analysis included 84 patients (42 HD, 42 DD). Comparative seroconversion rates are shown in Fig. 2 and Fig. 3. Seroconversion to at least 1 strain was significantly greater in the HD group (95.2%) compared to the DD group (69%) (P = 0.002) while the absolute difference in response rate was 0.26 (95% CI, 0.12–0.40). Similarly, seroconversion to at least two vaccine antigens was significantly higher in the HD group; however, this difference was not observed when assessing responses across three or four strains. For individual strains—A/H1N1, A/H3N2, B/Victoria, and B/Yamagata-seroconversion rates were higher in the HD group compared to the DD group: 69.1% versus 52.4%, 52.4% versus 38.1%, 52.4% versus 42.9%, and 61.9% versus 40.5%, respectively (P = 0.118, P = 0.188, P = 0.382, and P = 0.049). For B/Yamagata strain, the HD group had a significantly greater seroconversion rate when compared to DD group (P = 0.049) (Fig. 3).

**Fig. 2:**
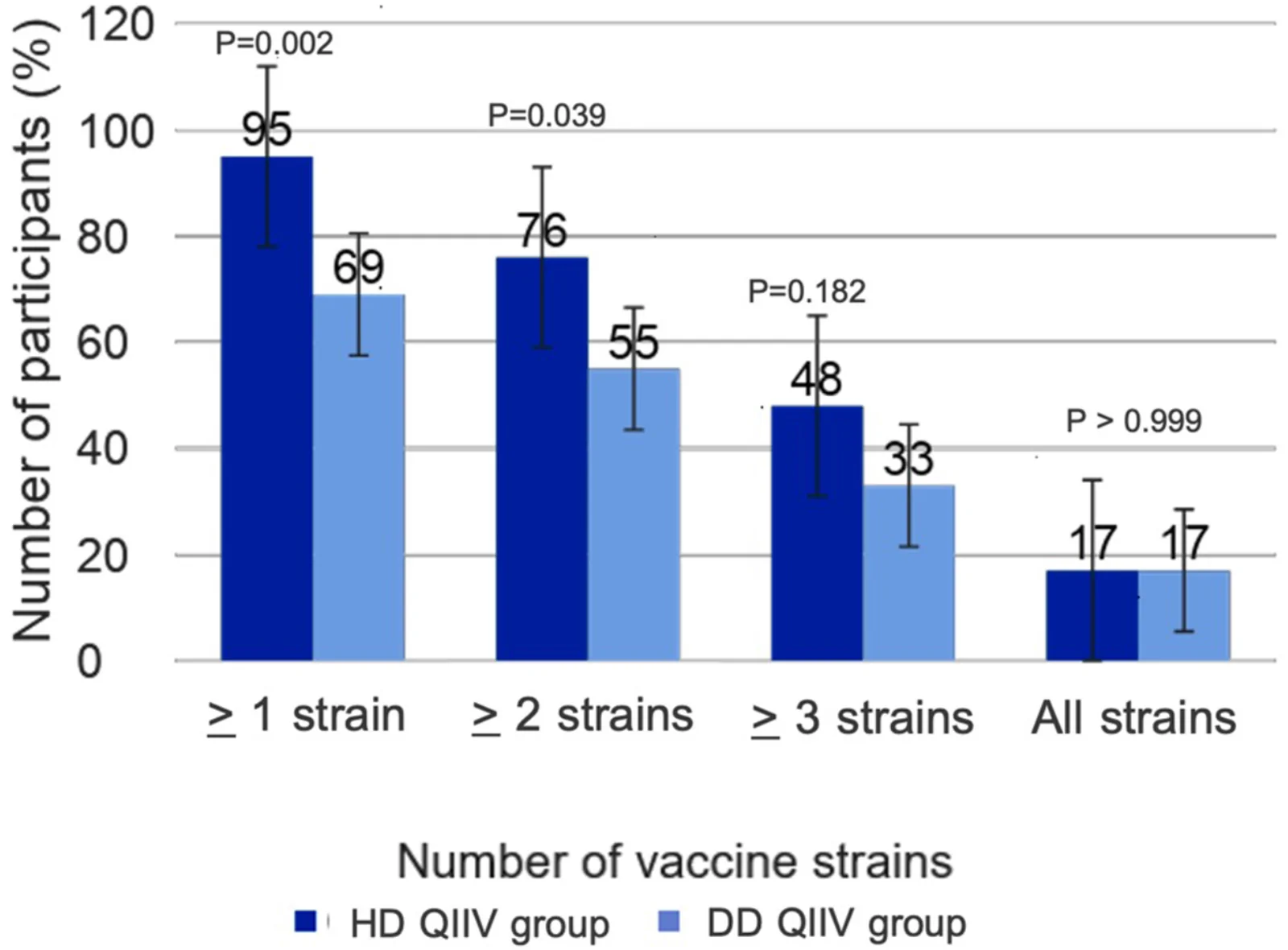
Seroconversion rates to at least 1, at least 2, at least 3 or all strains vaccine antigens with quadrivalent inactivated influenza vaccine.

**Fig. 3:**
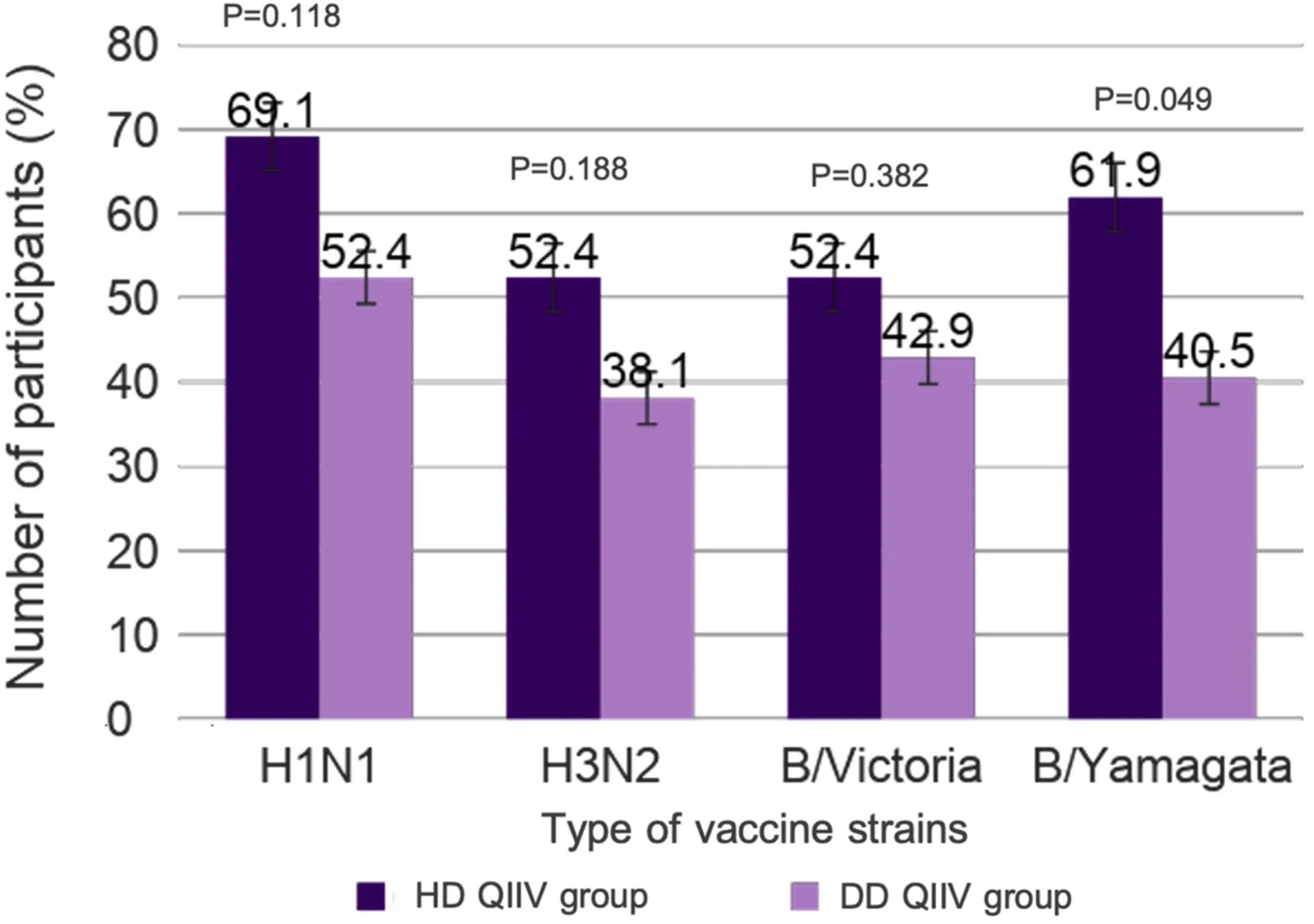
Seroconversion rates to each strain with quadrivalent inactivated influenza vaccine.

Both the HD and DD vaccines elicited robust immune responses across all four influenza strains (A/H1N1, A/H3N2, B/Victoria, and B/Yamagata), with significant increases in geometric mean titers (GMTs) from baseline (Supplementary Table S1). Although post-immunization GMTs appeared higher across all strains in the HD group, the differences were not statistically significant (Supplementary Table S1). Similarly, post-immunization seroconversion rates ranged from 52.4% to 69.1% in the HD group and from 38.1% to 52.4% in the DD group. Seroprotection rates ranged from 64.3% to 83.3% in the HD group and from 54.8% to 73.8% in the DD group (Supplementary Table S2).

Factors that affected vaccine SC to at least 1 strain were analyzed. In univariate analysis, patients who received HD vaccine (compared with DD vaccine) (OR 8.97, 95% CI [1.88,42.82], P = 0.006) and who vaccinated after 1 year post KT (OR 12.18, 95% CI [1.98–74.67], P = 0.007) had a greater likelihood of seroconversion. In multivariate analysis, Receiving HD vaccine (compared with DD vaccine) was an independent factor significantly associated with seroconversion to at least 1 strain (OR 17.59, 95% CI [2.10, 147.22], P = 0.008), as was receiving the vaccine more than 1 year post-KT (OR 34.76, 95% CI [2.59–454.45], P = 0.007) (Table 2).

**Table 2:** Factors associated with seroconversion to at least 1 Influenza antigen.

Clinical characteristics	Univariate analysis		Multivariate analysis	
	OR (95% CI)	P-value	OR (95% CI)	P-value
Age (per 1 year)	1.03 (0.97, 1.08)	0.346		
Female sex	2.12 (0.61, 7.31)	0.236		
Time from transplant (per 1 month)	1.003 (0.992, 1.014)	0.613		
Vaccinate after 1 year post-transplant	12.18 (1.98, 74.67)	0.007	34.28 (2.59,454.45)	0.007
Previous rejection	0.62 (0.06, 6.43)	0.688		
High-dose quadrivalent inactivated influenza vaccine (compared with double-dose vaccine)	8.97 (1.88, 42.82)	0.006	17.59 (2.10, 147.22)	0.008
Deceased donor kidney transplant	1.04 (0.33, 3.24)	0.950		
Tacrolimus	0.46 (0.09, 2.26)	0.340		
Cyclosporine	2.12 (0.43, 10.38)	0.352		
Mycophenolate mofetil	0.64 (0.18, 2.22)	0.482		
Mycophenolic sodium	1.11 (0.28, 4.45)	0.882		
Everolimus	1.09 (0.12, 10.11)	0.937		
Absolute lymphocyte count (per 100 cells/μl)	1.0007 (0.9998, 1.0016)	0.127		

Seroprotection rates for each strain with quadrivalent inactivated influenza vaccine showed a non-statistically significant trend toward higher seroprotection in the HD group compared to the DD group (Fig. 4).

**Fig. 4:**
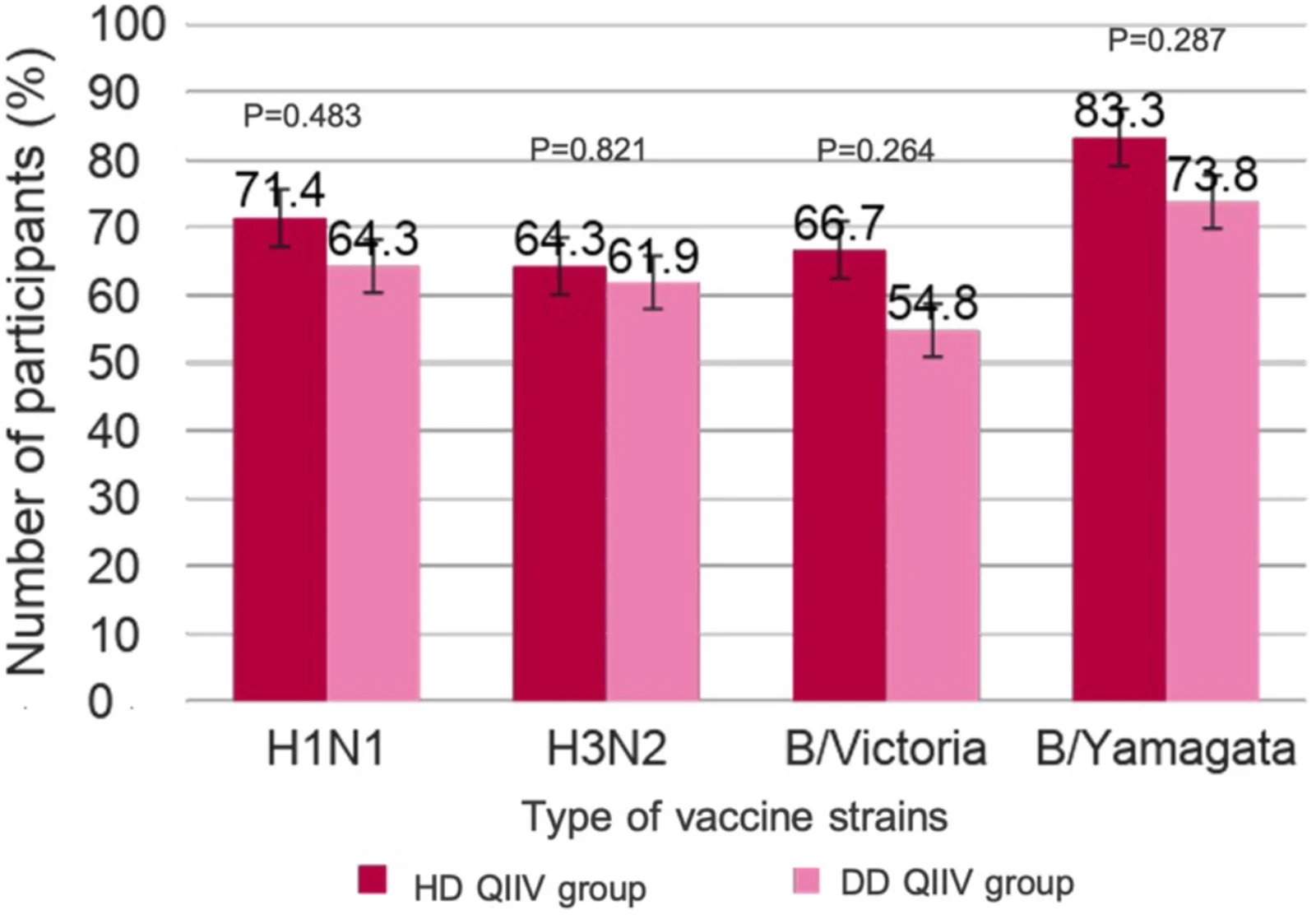
Seroprotection rates to each strain with quadrivalent inactivated influenza vaccine.

For subgroup analyses, among patients receiving mycophenolate mofetil less than 2 g per day, the HD vaccine was independently associated with higher seroconversion to at least one strain (OR 8.98, 95% CI 1.02–78.76; P = 0.047). In contrast, among patients with absolute lymphocyte counts less than 2000 cells/μL, the HD vaccine was not independently associated with higher seroconversion (OR 7.60, 95% CI 0.84–69.13; P = 0.072). Across subgroups, receiving the vaccine within 1 year post-KT remained significantly associated with lower seroconversion rates (Supplementary Table S3).

To assess effect modification, an interaction between vaccine type and time post-transplant was included in the multivariable model. Among patients vaccinated more than 1 year post-KT, the HD vaccine was associated with higher seroconversion than the DD vaccine (OR 13.10, 95% CI 1.59–108.26; P = 0.017), whereas no difference was observed within the first year post-KT (OR 0.69, 95% CI 0.06–8.45; P = 0.771; P for interaction = 0.0054).

Adverse events (AEs) after vaccination were assessed. There were no serious local or systemic AEs within 30 min after HD vaccination. Solicited AEs within 3 and 7 days after HD group were shown and compared with those after DD group in Fig. 5 and Supplementary Table S4. Within 3 days of vaccination, there were significantly fewer occurrences of pain at the injection site and muscle ache in the HD group compared to the DD group, with the following percentages: 4.8% versus 19.0%, and 4.8% versus 9.5% (P = 0.040 and P = 0.043, respectively). Most of the solicited adverse events were mild and self-limited (grade 1). On day 7, the AEs were almost entirely resolved. All AEs were graded as mild (grade 1). No acute rejection episodes occurred in vaccinated KT recipients. No unsolicited AEs were reported.

**Fig. 5:**
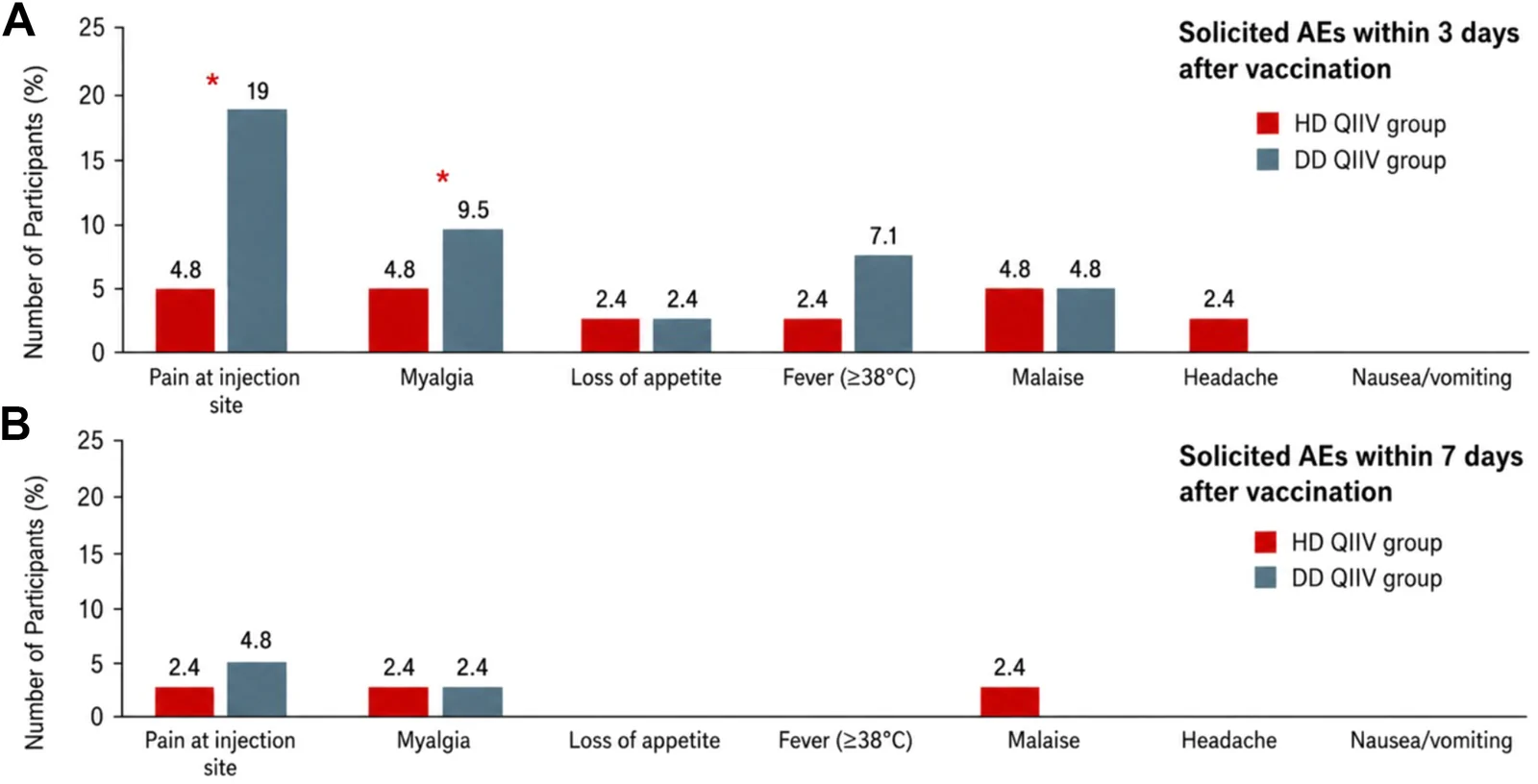
Solicited local and systemic adverse events on day-3 (A) and day-7 (B) after vaccination.

## Discussion

This study evaluated immune responses to compare the immunogenicity and safety of the 2023 southern hemisphere HD QIIV versus the DD QIIV in adult KT recipients, utilizing immunogenicity as a surrogate marker for protection against seasonal influenza. Nearly 95% of individuals who received the HD vaccine achieved seroconversion to at least one strain, compared with 69% in the DD group. This indicates that the HD vaccine elicited a substantially stronger immune response, translating to an approximately 26% higher response rate overall. The 95% confidence interval (12%–40%) further supports the robustness of this effect and suggests that the improvement in immunogenicity is clinically meaningful, while also being associated with a comparatively lower frequency of adverse reactions than the DD vaccine. In the multivariate analysis, Receiving HD vaccine was independently associated with seroconversion to at least 1 vaccine antigen. In addition, receiving the vaccine more than 1 year post-transplant were also significantly associated with seroconversion rates.

In contrast, post-vaccination GMTs and seroprotection rates showed no significant differences between the two groups. This may be attributed to high pre-existing baseline titers present in both cohorts. The lack of statistical significance in GMTs likely reflects the limited power of small sample size to detect subtle shifts in absolute magnitude, even when a significant proportion of the HD group met the threshold for a protective response. These findings suggest that although both higher-dose strategies increase antibody levels, the HD formulation more consistently achieves the threshold required for seroconversion in KT recipients.

KT recipients generally have poor responses to influenza vaccine due to receiving life-long immunosuppression. A single dose of monovalent 2009 influenza A (H1N1) vaccine resulted in poor immunogenicity among adult KT recipients that only 31.9% and 70% when compared to those with normal immune status.^2^ In additional studies, a trend toward increased immunogenicity was observed with a double-dose vaccine. Previous studies in transplant recipients have shown greater seroconversion rates at least 1 strain of influenza antigens with DD vaccine compared to SD vaccine at 40% versus 26%, respectively.^3^ Recently, Odongo FCA et al. performed a study to compare the immunogenicity of DD vaccine, SD vaccine and boosted dose (2 doses of SD vaccine in the same season) in KT recipients. The study showed that there was improved seroconversion for A/H1N1 2009 strain in the group that received DD vaccine and boosted dose compared to SD vaccine at 56%, 60% and 10%, respectively.^4^ However, two-visit requirement strategies for vaccination may reduce completion rates.

HD influenza vaccine was approved by the US Food and Drug Administration in 2009 and HD QIIV was recently licensed in Thailand in 2022. However, evidence for a benefit of these alternative vaccination strategies specifically in the transplant population is weaker, relying only on small clinical trials. The American Society for Transplantation recommends that high-dose or two boosted standard doses administered one month apart provide greater immunogenicity than a single standard dose and may be preferred over standard dosing.^6^ Prior study in organ transplant recipients that used the HD influenza vaccine in 166 SOT recipients by Natori Y et al. showed that vaccine responses were significantly higher in the high-dose (79%) as compared to the standard-dose vaccine group (53%).^5^ Additionally, Mombelli M. et al. recently demonstrated that high-dose influenza vaccines elicited stronger immunogenic responses (66%) compared to standard-dose vaccines (42%), with outcomes varying by transplant organ type and timing post-transplant.^8^ Similarly, our study demonstrated that HD vaccine had significantly greater seroconversion rates at least 1 strain compared to DD vaccine (95.2% versus 69%, respectively).

Vaccine subresponsiveness remains a significant challenge across various immunocompromised cohorts. In patients living with HIV, randomized trials have established that HD vaccines significantly improve seroprotection and seroconversion rates compared to standard doses, particularly in individuals with lower CD4+ T-cell counts (<200 cells/μL).^9^ Similarly, in oncology patients undergoing chemotherapy, ‘enhanced’ strategies—such as double doses or boosters—have been shown to improve serological responses. However, the high cost and limited availability of specialized HD formulations frequently necessitate the use of DD strategies in clinical practice.^10^ By conducting this trial in KT recipients, we provide critical data that may not only refine transplant-specific protocols but also contribute to the broader understanding of dose-optimization across the spectrum of immunocompromised health.

Factors associated with seroconversion have been previously reported in the literature. In particular, a longer interval from transplantation has been identified as a favorable predictor of seroconversion in adult kidney transplant recipients receiving COVID-19 vaccination, likely due to reduced intensity of immunosuppression in the late post-transplant period.^11^ This finding aligns with the seroconversion rates observed in our cohort of kidney transplant recipients who received the high-dose influenza vaccine. Additionally, lower doses of immunosuppressants—such as mycophenolate mofetil at less than 2 g per day—have been associated with improved seroconversion in adult solid organ transplant recipients in prior studies.^5^ However, this association was not observed in our cohort.^6^ In subgroup analyses, among patients receiving low-dose mycophenolate mofetil less then 2 g per day, the HD vaccine was independently associated with higher seroconversion. In contrast, this association was not observed in those with absolute lymphocyte counts less than 2000 cells/μL, likely reflecting impaired immune responsiveness. Vaccination within the first year post-transplant was consistently associated with lower seroconversion across subgroups, underscoring the impact of early post-transplant immunosuppression. These findings suggest that, although HD vaccination may enhance responses in selected patients, host factors remain key determinants of vaccine immunogenicity. Interaction analysis showed that the advantage of the HD vaccine was most evident beyond the first year post-transplant, with a ∼13-fold higher odds of seroconversion compared with DD. In contrast, no benefit was observed within the first year, likely reflecting limited vaccine responsiveness during intensive immunosuppression. These findings suggest that the relative benefit of HD QIIV is greatest in the stable, maintenance phase post-transplantation.

Overall, the HD vaccine was found to be safe and well-tolerated. As previously reported, the HD vaccine exhibited a non-significant trend of tenderness at the injection site compared to the standard-dose (SD) vaccine in SOT recipients, with approximately one fourth of patients in each group experiencing this symptom.^5^ However, our study’s findings contrast with this, as only 5% of patients receiving the HD vaccine reported localized pain, a substantially lower incidence compared to those who received the DD vaccine. This discrepancy may be attributed to the lack of a commercial formula for the DD vaccine, which requires two shots in each deltoid. Notably, most adverse events were mild and resolved spontaneously in our study.

The strength of this study lies in being one of the few to investigate the immunogenicity of the HD QIIV among adult KT recipients. We assessed immunogenicity using the HI assay, a widely used method for measuring antibody titers in clinical trials. Additionally, we evaluated short-term solicited adverse events following vaccination. Although this single-center study was conducted in Thailand, the findings are globally relevant as the participants received standard immunosuppressive regimens.^4^^,^^6^ Thus, these immunologic responses likely extend to other transplant populations. Furthermore, in many low- and middle-income countries where HD vaccines are often unavailable or cost-prohibitive, we evaluated evidence-based alternative to improve protection in vulnerable populations. However, the study has several limitations. First, the open-label design may introduce bias. While the primary outcome focused on vaccine immune response, the seroconversion and seroprotection immunogenicity titers may not be directly applicable to immunocompromised patients due to their altered immune profiles^12^ Furthermore, the study did not assess cell-mediated immune responses, which would have provided a more comprehensive understanding of vaccine-induced immunity.^13^ Additionally, the clinical efficacy of the vaccine was not evaluated, which would have required a larger sample size and the modest sample size may have limited the power to detect rare adverse events or subtle differences in vaccine response. The protective effect of this vaccine regimen remains inconclusive due to a lack of studies on its clinical efficacy. However, immunogenicity is widely recognized as a surrogate marker for protection, and immunogenicity endpoints are commonly utilized for the licensure of seasonal influenza vaccines.^14^ In real-world settings, giving two separate injections of the SD vaccine at the same visit is impractical, especially since no commercial DD formulation exists. Finally, while short-term adverse events were documented, long-term safety and potential indirect adverse effects warrant further investigation.

In conclusion, in this study, HD QIIV vaccine demonstrated significantly better immunogenicity with comparatively reduced occurrence of adverse reactions in adult SOT recipients. These results indicate that, where accessible, HD QIIV could be considered a more suitable option for this patient population.

## Contributors

JB conceptualized the study, developed the study instruments, super-vised fieldwork, verified the data, and interpreted the findings. TA, KB, and JB led the statistical analysis. TA and JB had full access to all study data and took responsibility for the integrity of the data and the accuracy of the data analysis. TA and JB drafted the first version of the manuscript. TA, KB, SK, SB, SK, and JB critically revised the manuscript for important intellectual content. TA and JB contributed to study design, field implementation, and interpretation of findings, with particular attention to cultural relevance, ethical considerations, and contextual appropriateness. All authors reviewed and approved the final version of the manuscript and take responsibility for its content.

## Data sharing statement

The data underlying the results reported in this paper will be available, after deidentification, from the corresponding authors on reasonable request. Data will be provided to researchers who provide a methodologically sound proposal to enhance the aims of the study, approved by an independent review board.

## Declaration of generative AI and AI-assisted technologies in the writing process

During the preparation of this manuscript, the authors used ChatGPT-4o to assist with English language editing. The authors thoroughly reviewed and revised the content following its use and take full responsibility for the final version of the manuscript.

## Declaration of interests

Authors do not have any conflicts of interest to declare.

## Appendix A Supplementary data

Thanachote protocol HD flu vaccine in KT

Supplementary Fig. S1 Supplementary Fig. S1. Assessment sheet for immediate adverse events after vaccination.

Supplementary Fig. S2 Supplementary Fig. S2. Assessment sheet for solicited adverse events after vaccination.
