# Supplementary material for: Immunogenicity and safety of high-dose versus double-dose quadrivalent inactivated influenza vaccine in adult kidney transplant recipients: a randomized controlled trial (2023)
Source: Lancet Reg Health Southeast Asia. 2026 Sep 25;55:100873. doi: 10.1016/j.lansea.2026.100873 (PMC13634540; doi:10.1016/j.lansea.2026.100873)
Supplement: Supplementary file 1 [file mmc2.pdf]

Assessment of AE after vaccination 30 min. (Immediate AEs)

Did any Solicited adverse event occurs within 30 minutes after vaccination?

1) Solicited local injection site reactions:

Pain	☐ None ☐ Grade1 (mild) ☐ Grade2 (severe without action) ☐ Grade3 (severe with action)
Redness	☐ None ☐ Yes, ______mm
Swelling.	☐ None ☐ Yes, ______mm

Action Taken: ☐ None ☐ Medication ☐ Health Care Provider Contact ☐ Hospitalized
☐ Discontinuation of Study vaccination

2) Solicited systemic adverse events

Headache	☐ None ☐ Grade1 (mild) ☐ Grade2 (severe without action) ☐ Grade3 (severe with action)
Malaise	☐ None ☐ Grade1 (mild) ☐ Grade2 (severe without action) ☐ Grade3 (severe with action)
Myalgia	☐ None ☐ Grade1 (mild) ☐ Grade2 (severe without action) ☐ Grade3 (severe with action)

Action Taken: ☐ None ☐ Medication ☐ Health Care Provider Contact ☐ Hospitalized
☐ Discontinuation of Study vaccination

Note:____

____

Investigator's initial: ______________________________ **Date:** ____/_____/______
