# Supplementary material for: Immunogenicity and safety of high-dose versus double-dose quadrivalent inactivated influenza vaccine in adult kidney transplant recipients: a randomized controlled trial (2023)
Source: Lancet Reg Health Southeast Asia. 2026 Sep 25;55:100873. doi: 10.1016/j.lansea.2026.100873 (PMC13634540; doi:10.1016/j.lansea.2026.100873)
Supplement: Supplementary file 2 [file mmc3.pdf]

Phone call for solicited adverse events

First name ____________________ Last name ___________________

First dose date _____________________

Second dose date _____________________

	Dose	Date	Time	Symptom		Date of relief	Medication and dosing	Date of treatment	Date of discontinuation	Investigator's initial
**Day 3**	1			**-pain at injection site**<br>☐ mild<br>☐ severe without medication<br>☐ severe with medication<br>**-redness at injection site**<br>☐ yes size______mm.<br>**-swelling at injection site**<br>☐ yes size______mm.	**-headache**<br>☐ mild<br>☐ severe without medication<br>☐ severe with medication<br>**-malaise**<br>☐ mild<br>☐ severe without medication<br>☐ severe with medication<br>**-myalgia**<br>☐ mild<br>☐ severe without medication<br>☐ severe with medication					
**Day 7**	1			**-pain at injection site**<br>☐ mild<br>☐ severe without medication<br>☐ severe with medication<br>**-redness at injection site**<br>☐ yes size______mm.<br>**-swelling at injection site**<br>☐ yes size______mm.	**-headache**<br>☐ mild<br>☐ severe without medication<br>☐ severe with medication<br>**-malaise**<br>☐ mild<br>☐ severe without medication<br>☐ severe with medication<br>**-myalgia**<br>☐ mild<br>☐ severe without medication<br>☐ severe with medication					
