# Supplementary material for: Immunogenicity and safety of high-dose versus double-dose quadrivalent inactivated influenza vaccine in adult kidney transplant recipients: a randomized controlled trial (2023)
Source: Lancet Reg Health Southeast Asia. 2026 Sep 25;55:100873. doi: 10.1016/j.lansea.2026.100873 (PMC13634540; doi:10.1016/j.lansea.2026.100873)
Supplement: Supplementary file 3 [file mmc1.docx]

**Study protocol**

**Title**

Immunogenicity and safety of high-dose versus double-dose quadrivalent inactivated influenza vaccine in adult kidney transplant recipients:

A randomized controlled trial (HANUMAN Study)

**Protocol Number**

MURA2023/309 Version 2.0 July 11, 2023

**Approved by**

The Institutional Review Board of Faculty of Medicine Ramathibodi Hospital,

Mahidol University, Bangkok, Thailand

**Investigators**

Thanachote Arayanumas, MD^1^

Kobporn Boonnak, PhD^2^

Surasak Kantachuvesiri, MD^3,4^

Sarinya Boongird, MD^3,4^

Sasisopin Kiertiburanakul, MD, MHS^5^

Jackrapong Bruminhent, MD^4,5^

^1^Department of Medicine, Faculty of Medicine Ramathibodi Hospital,

Mahidol University, Bangkok, Thailand

^2^Department of Immunology, Faculty of Medicine Siriraj Hospital,

Mahidol University, Bangkok, Thailand

^3^Division of Nephrology, Department of Medicine, Faculty of Medicine Ramathibodi Hospital, Mahidol University, Bangkok, Thailand

^4^Ramathibodi Excellence Center for Organ Transplantation, Faculty of Medicine Ramathibodi Hospital, Mahidol University, Bangkok, Thailand

^5^Division of Infectious Diseases, Department of Medicine, Faculty of Medicine Ramathibodi Hospital, Mahidol University, Bangkok, Thailand

**1. BACKGROUND**

Influenza infection is known to be an important cause of morbidity and mortality around the world especially in solid organ transplant recipients. Complications of influenza infection, including viral and bacterial pneumonia, appear to be common in this patient population. Therefore, omitting from Influenza infection is recommended.

American Society for Transplantation/IDCOP Guideline 2019 has recommended that high-dose or booster dosing in the same season has greater immunogenicity over a single standard dose and may be preferred over standard dosing^6^. Although both high-dose (HD) and standard-dose booster regimens improve immunogenicity, a two-visit requirement for vaccination may reduce completion rates.

A high-dose (HD) influenza vaccine, containing 60 μg from 3 circulating strain, represents a 4-fold increase in dose compared to the standard vaccine (SD). Previous studies have shown that both high-dose (HD) and double-dose (DD) inactivated influenza vaccines elicit a stronger immune response in solid organ transplant (SOT) recipients compared to standard-dose (SD) vaccines. However, a direct comparison between high-dose (HD) and double-dose (DD) quadrivalent inactivated influenza vaccines (QIIV) has not yet been explored. To assess this, we performed a randomized trial to compare the safety and immunogenicity of HD vs DD influenza vaccine in adult kidney transplant patients.

**2. RATIONALE**

Annual influenza immunization is recommended for all SOT recipients. However, immunogenicity—the ability to generate an adequate immune response— is known to be suboptimal response than normal host due to receiving lifelong immunosuppression.^1,2^. We aimed to investigate the immunogenicity and safety of high-dose quadrivalent inactivated influenza vaccine (HD QIIV) versus Double-dose (DD QIIV) in adult kidney transplant (KT) patients.

**3. STUDY AIM & HYPOTHESIS**

**3.1 Aim**

To examine the immunogenicity and safety of high dose (HD) versus double-dose (DD) quadrivalent inactivated influenza vaccine (QIIV) in SOT recipients.

**3.2 Hypothesis**

A high-dose (HD) of influenza vaccine could potentially elicit a greater immune response in SOT recipients when compared to Double-dose (DD) quadrivalent inactivated influenza vaccine

**4. STUDY OBJECTIVE**

**4.1 Primary objective**

To assess the seroconversion for at least 1 viral strain of high dose (HD) versus double-dose (DD) quadrivalent inactivated influenza vaccine (QIIV) in adult KT recipients.

**4.2 Secondary objectives**

To investigate the seroconversion and seroprotection of HD QIIV versus SD QIIV in adult KT recipients

To determine factors associated with seroconversion and seroprotection

To assess the safety of HD QIIV and SD QIIV in adult KT recipients.

**5. METHODOLOGY**

**5.1 Study site**

Ramathibodi Hospital, Mahidol University, Bangkok, Thailand

**5.2 Study design**

An open-label randomized controlled trial will be performed at Ramathibodi Hospital, Mahidol University, Bangkok, Thailand. We will enroll adult KT recipients. Participants will be randomized to receive either HD or DD influenza vaccine in a 1:1 ratio to receive either HD vaccine (0.7mL of HD QIIV, Efluelda, Sanofi, 60 μg per strain) or DD vaccine (2 doses of 0.5 mL of SD QIIV, Fluarix Tetra, Sanofi, 15 μg per strain) via intramuscular injection in the deltoid muscle of the arm. All participants will undergo a blood collection (4 mL) prior to additional vaccination and a follow-up sample (4 mL) will be collected four weeks after immunization to measure their immunogenicity

The recruitment and consent process are as the following: The study coordinator will contact all KT participants. A telephone script will be used to introduce this study and initiate the consent process. Questions about the study can be answered over the phone at this time. The study investigator(s) will be available in case of questions. Study eligibility and screening for inclusion and exclusion criteria will take place. Consent will then be provided on site at Ramathibodi Hospital in-person.

There will be two visits during the study, the first visit will be when the participant receives the first dose either HD or DD QIIV after the participant has consented to take part in the study. Every participant will have their blood drawn for the initial sample on the day they receive the vaccine. All participants will be scheduled for the second visit at four weeks after receiving the vaccine for the second blood collection.

**5.3 Study intervention**

HD vaccine (0.7mL of HD QIIV, Efluelda, Sanofi, 60 μg per strain) and DD vaccine (2 doses of 0.5 mL of SD QIIV, Fluarix Tetra, Sanofi, 15 μg per strain) will be obtained and administered at Ramathibodi Hospital and 30 minute observation will be performed as per standard practice.

**5.4 Study population**

**Inclusion criteria**

- Adult KT recipients (age > 15 years old)
- Patients who are at least 3 months after transplant & stable in terms of allograft function and immunosuppressive regimen
- Patients can return to the hospital on day 28 after vaccination

**Exclusion criteria**

- Patients who receive influenza vaccination within 6 months
- Patients with a history of severe allergy to previous influenza vaccine
- Concurrent infection or rejection

**5.5 Study Follow-up**

All participants will receive vital sign assessment and a physical examination before receiving their vaccination. Following administration, they will be monitored for immediate adverse events (AEs) for up to 30 minutes **(figure 1)**, including both local and systemic reactions. To track post-vaccination effects, solicited AEs will be assessed via telephone follow-ups on days 3 **(figure 2)** and 7**(figure 3)**, while participants will be encouraged to document and report any unsolicited AEs in their symptom diaries. The study team reviewed all reported AEs to determine potential causal associations with vaccination. Additionally, participants will be advised to notify the research team of any suspected complication and instructed to visit the study facility for further assessment and management.

**Figure 1.** Assessment sheet of immediate adverse events after vaccination

**Figure 2.** Assessment sheet of solicited adverse events after vaccination

**Figure 3.** Assessment sheet of unsolicited adverse events after vaccination

**5.6 Patient Confidentiality**

Participants will be randomly assigned in a 1:1 ratio to receive either HD QIIV or DD QIIV. All samples will be kept until the study is complete.

The trial site will preserve all study-related files and documents for a maximum of 10 years after the study has been finalized. All document records will be securely stored in a locked filing cabinet within a restricted-access office. Baseline demographic data will be collected and recorded in an electronic database, which will be securely maintained. Access to the database will be restricted to authorized personnel and protected by password authentication to ensure data confidentiality.

**5.7 Laboratory methods**

**5.7.1 Hemagglutination Inhibition (HAI) Assay**

4 ml of peripheral blood will be collected via venipuncture and centrifuged for 10-mins to obtain the serum. Serum samples were serially diluted two-fold in V-bottom 96-well microtiter plates, starting from a 1:10 dilution. Each well contained 25 µL of diluted serum. An equal volume (25 µL) of standardized influenza virus antigen (4 HAU/50 µL) according to WHO was added to each well and incubated for 30 minutes at room temperature to allow antigen-antibody interactions. The test used reference influenza strains corresponding to the vaccine components:

- A/Sydney/5/2021 (H1N1)pdm09-like virus
- A/Darwin/9/2021 (H3N2)-like virus
- B/Austria/1359417/2021 (B/Victoria lineage)-like virus
- B/Phuket/3073/2013 (B/Yamagata lineage)-like virus

The HAI titer was defined as the highest dilution of serum that completely inhibited hemagglutination. And defining by WHO:

- **Seroconversion** was defined as both four-fold increase in HAI titer from baseline reaching a seroprotective titer
- **Seroprotection** was defined as achieving a post-vaccination titer of ≥1:40
- **Geometric mean titer** was defined as average antibody titer for a group of subjects

**5.8 Outcome**

**5.8.1. Primary outcome:**

The primary outcome will be the assessment of seroconversion at least 1 strain in HD QIIV compared to DD QIIV in adult KT recipients.

**5.8.2 Secondary outcome:**

The secondary outcome will be the assessment of the seroconversion, seroprotection, factors that could determine a better immunogenicity, and the safety profile of the in High-dose QIIV compared to Double-dose QIIV in adult KT recipients.

**6 STATISTICAL METHODS**

**6.1 Sample size calculation**

We estimated that the seroconversion rate in the group receiving the high-dose influenza vaccine is approximately 80% to at least 1 strain^4^, while the seroconversion rate in the group receiving the double-dose influenza vaccine is expected to be 50%. Using a statistical power of 80% and a significance level (α) of 0.05, the required sample size is calculated to be 40 participants per group, at 1:1 randomization, totaling 88 participants is required considering a drop-out rate of 10%.

**6.2 Statistical analyses**

- Data will be described using mean and standard deviation (SD) or median and inter quartile range (IQR) as appropriate for continuous variables, and percentage for categorical variables.
- The corresponding data will be compared between groups using t-test (or Wilcoxon rank-sum test where appropriate) and Chi-square test (or Fisher-Exact where appropriate).
- A univariate, multivariate will use logistic regression and estimated odd ratio (OR) with 95% confidence interval (CI) for primary outcome.
- Variables whose p value were < 0.05 in this step were simultaneously considered in a multivariate, only significant variables suggested by a likelihood ratio (LR) test were kept in the final model.
- Study groups will be compared for the secondary outcome which included a safety profile and predictors of immunogenicity. Adverse events (AE) in both HD and DD groups will be collected.

Local and systemic AE during the first 3 and 7 days will be reported using descriptive analysis and compared between groups.

- All statistical analyses will be performed using STATA version 18 (Stata Crop., College Station, Texas, USA).
- A P-value of <0.05 will be considered statistically significant.

**7 SIGNIFICANCE OF THE STUDY**

A direct assessment comparing HD QIIV versus DD QIIV with well-designed study as additional evidence to a group of series about how to enhanced immunity in SOT recipients. The study will be able to provide the new data and potentially apply it as an effective vaccination method for SOT recipients and other vulnerable immunocompromised patients.

**References**

1. Mombelli M, Kampouri E, Manuel O. Influenza in solid organ transplant recipients: epidemiology, management, and outcomes. Expert Rev Anti Infect Ther. 2020;18:103-12.

2. Watcharananan SP, Thakkinstian A, Srichunrasmee C, Chuntratita W, Sumethkul V. Comparison of the immunogenicity of a monovalent influenza A/H1N1 2009 vaccine between healthy individuals, patients with chronic renal failure, and immunocompromised populations. Transplant Proc. 2014;46:328-31.

3. Mombelli M, Rettby N, Perreau M, Pascual M, Pantaleo G, Manuel O. Immunogenicity and safety of double versus standard dose of the seasonal influenza vaccine in solid-organ transplant recipients: A randomized controlled trial. Vaccine. 2018;36:6163-9.

4. Odongo FCA, Braga PE, Palacios R, et al. An Open-label Randomized Controlled Parallel-group Pilot Study Comparing the Immunogenicity of a Standard-, Double-, and Booster-dose Regimens of the 2014 Seasonal Trivalent Inactivated Influenza Vaccine in Kidney Transplant Recipients.Transplantation. 2022;106:210-20.

5. Natori Y, Shiotsuka M, Slomovic J, et al. A Double-Blind, Randomized Trial of High-Dose vs Standard-Dose Influenza Vaccine in Adult Solid-Organ Transplant Recipients. Clin Infect Dis. 2018;66:1698-704.

6. Kumar D, Blumberg EA, Danziger-Isakov L, et al; AST Infectious Diseases Community of Practice. Influenza vaccination in the organ transplant recipient: review and summary recommendations. Am J Transplant. 2011;11:2020–30.

7. Gianchecchi E, Torelli A, Montomoli E. Hum Vaccin Immunother. 2019;15(5):1021-1030.
