# Supplementary material for: Immunogenicity and safety of high-dose versus double-dose quadrivalent inactivated influenza vaccine in adult kidney transplant recipients: a randomized controlled trial (2023)
Source: Lancet Reg Health Southeast Asia. 2026 Sep 25;55:100873. doi: 10.1016/j.lansea.2026.100873 (PMC13634540; doi:10.1016/j.lansea.2026.100873)
Supplement: Supplementary file 4 [file mmc4.docx]

**Supplementary Table 1.** Geometric mean titers of high-dose vs double-dose quadrivalent influenza vaccine, per-protocol populations

| **Geometric Mean Titers (median) (IQR)** | **Pre-**  **vaccination** | **Post-vaccination** | **P-value** | **Compared to DD QIIV post-vaccination** |
| --- | --- | --- | --- | --- |
| **HD QIIV (n=42)** |  |  |  |  |
| A/H1N1 | 10 (10, 15) | 160 (20, 640) | < 0.001 | 0.083 |
| A/H3N2 | 15 (10, 20) | 60 (20, 160) | < 0.001 | 0.286 |
| B/Victoria | 10 (10, 20) | 40 (20, 160) | < 0.001 | 0.380 |
| B/Yamagata | 40 (10, 80) | 160 (80, 320) | < 0.001 | 0.112 |
| **DD QIIV (n=42)** |  |  |  |  |
| A/H1N1 | 10 (10, 20) | 80 (10, 160) | < 0.001 | N/A |
| A/H3N2 | 15 (10, 40) | 40 (20, 160) | < 0.001 | N/A |
| B/Victoria | 10 (10, 30) | 40 (20, 320) | < 0.001 | N/A |
| B/Yamagata | 40 (20, 80) | 80 (40, 320) | < 0.001 | N/A |

HD QIIV, high-dose quadrivalent inactivated influenza vaccine; DD QIIV, double-dose quadrivalent inactivated influenza vaccine; IQR, interquartile range
