# Supplementary material for: Immunogenicity and safety of high-dose versus double-dose quadrivalent inactivated influenza vaccine in adult kidney transplant recipients: a randomized controlled trial (2023)
Source: Lancet Reg Health Southeast Asia. 2026 Sep 25;55:100873. doi: 10.1016/j.lansea.2026.100873 (PMC13634540; doi:10.1016/j.lansea.2026.100873)
Supplement: Supplementary file 5 [file mmc5.docx]

**Supplementary Table 2.** Seroconversion and seroprotection rates of high-dose vs double-dose quadrivalent influenza vaccine, per-protocol populations

| **Rate, N (%)** | **Total**  **(n = 84)** | **HD QIIV**  **(n = 42)** | **DD QIIV**  **(n = 42)** | **P-value** |
| --- | --- | --- | --- | --- |
| **Seroconversion** | | | | |
| H1N1 | 51 (60.7) | 29 (69.1) | 22 (52.4) | 0.118 |
| H3N2 | 38 (45.2) | 22 (52.4) | 16 (38.1) | 0.188 |
| B/Victoria | 40 (47.6) | 22 (52.4) | 18 (42.9) | 0.382 |
| B/Yamagata | 43 (51.2) | 26 (61.9) | 17 (40.5) | 0.049 |
| **Seroprotection** | | | | |
| H1N1 | 57 (67.9) | 30 (71.4) | 27 (64.3) | 0.483 |
| H3N2 | 53 (63.1) | 27 (64.3) | 26 (61.9) | 0.821 |
| B/Victoria | 51 (60.7) | 28 (66.7) | 23 (54.8) | 0.264 |
| B/Yamagata | 66 (78.6) | 35 (83.3) | 31 (73.8) | 0.287 |

HD QIIV, high-dose quadrivalent inactivated influenza vaccine; DD QIIV, double-dose quadrivalent inactivated influenza vaccine
