# Supplementary material for: Immunogenicity and safety of high-dose versus double-dose quadrivalent inactivated influenza vaccine in adult kidney transplant recipients: a randomized controlled trial (2023)
Source: Lancet Reg Health Southeast Asia. 2026 Sep 25;55:100873. doi: 10.1016/j.lansea.2026.100873 (PMC13634540; doi:10.1016/j.lansea.2026.100873)
Supplement: Supplementary file 6 [file mmc6.docx]

**Supplementary Table 3.** Subgroup analysis of factors associated with seroconversion to at least 1 Influenza antigen

| **Mycophenolate mofetil**  **< 2 g/day** | **Univariate** | | **Multivariate** | |
| --- | --- | --- | --- | --- |
|  | **OR (95% CI)** | **P-value** | **OR (95% CI)** | **P-value** |
|  |  |  |  |  |
| HD QIIV | 5.00 (0.95, 25.71) | 0.057 | 8.98 (1.02, 78.76) | 0.047 |
| DD QIIV | 1 |  | 1 |  |
| Receiving the vaccine more than 1 year post-KT | **14.29** (1.41,100.00) | 0.025 | **33.33** (1.79, 100.00) | 0.019 |
| Receiving the vaccine within 1 year post-KT | 1 |  | 1 |  |
| **Absolute lymphocyte count**  **< 2000 cells/µL** | **Univariate** | | **Multivariate** | |
|  | **OR (95% CI)** | **P-value** | **OR (95% CI)** | **P-value** |
|  |  |  |  |  |
| HD QIIV | 4.25 (0.78, 23.11) | 0.094 | 7.60 (0.84, 69.13) | 0.072 |
| DD QIIV | 1 |  | 1 |  |
| Receiving the vaccine more than 1 year post-KT | **14.29** (1.23,100.00) | 0.033 | **25.00** (1.49, 100.00) | 0.026 |
| Receiving the vaccine within 1 year post-KT | 1 |  | 1 |  |

HD QIIV, high-dose quadrivalent inactivated influenza vaccine; DD QIIV, double-dose quadrivalent inactivated influenza vaccine
