# Supplementary material for: Immunogenicity and safety of high-dose versus double-dose quadrivalent inactivated influenza vaccine in adult kidney transplant recipients: a randomized controlled trial (2023)
Source: Lancet Reg Health Southeast Asia. 2026 Sep 25;55:100873. doi: 10.1016/j.lansea.2026.100873 (PMC13634540; doi:10.1016/j.lansea.2026.100873)
Supplement: Supplementary file 7 [file mmc7.docx]

**Supplementary Table 4.** Solicited local and systemic adverse events on day-3 and day-7 after vaccination

| **Adverse events** | **Total**  **(n = 84)** | **HD QIIV**  **(n = 42)** | **DD QIIV**  **(n = 42)** | **P-value** |
| --- | --- | --- | --- | --- |
| **Post-vaccine day 3** |  |  |  |  |
| **Pain at the injection site** | 10 (11.9) | 2 (4.8) | 8 (19.0) | 0.040 |
| **Myalgia** | 6 (7.1) | 2 (4.8) | 4 (9.5) | 0.043 |
| **Loss of appetite** | 2 (2.4) | 1 (2.4) | 1 (2.4) | 1.000 |
| **Fever** | 4 (4.8) | 1 (2.4) | 3 (7.1) | 0.616 |
| **Malaise** | 4(4.8) | 2(4.8) | 2(4.8) | 1.000 |
| **Headache** | 1(1.2) | 1(2.4) | 0 (0.0) | 0.116 |
| **Nausea/vomiting** | 0 (0.0) | 0 (0.0) | 0 (0.0) | NA |
| **Others** | 2 (2.4) | 1 (2.4) | 1 (2.4) | 1.000 |
| **Post-vaccine day 7** |  |  |  |  |
| **Pain at the injection site** | 3 (3.6) | 1 (2.4) | 2 (4.8) | 0.210 |
| **Myalgia** | 2 (2.4) | 1 (2.4) | 1 (2.4) | 1.000 |
| **Loss of appetite** | 0 (0.0) | 0 (0.0) | 0 (0.0) | NA |
| **Fever** | 0 (0.0) | 0 (0.0) | 0 (0.0) | NA |
| **Malaise** | 1(1.2) | 1(2.4) | 0(0.0) | 0.116 |
| **Headache** | 0 (0.0) | 0 (0.0) | 0 (0.0) | NA |
| **Nausea/vomiting** | 0 (0.0) | 0 (0.0) | 0 (0.0) | NA |
| **Others** | 0 (0.0) | 0 (0.0) | 0 (0.0) | NA |

HD QIIV, high-dose quadrivalent inactivated influenza vaccine; QIV-DD, double-dose quadrivalent inactivated vaccine
